# Frequency-dependent adaptations in energy expenditure, strength, mood, and inflammation following 16 to 32 weeks of combined aerobic and resistance training in older women

**DOI:** 10.1007/s00421-026-06161-5

**Published:** 2026-03-24

**Authors:** Abdelrahman M. Elshakankiry, Gary R. Hunter, Cátia Martins, Gordon Fisher

**Affiliations:** 1 Department of Nutrition Sciences, University of Alabama at Birmingham (UAB), Birmingham, AL 35294-3360 USA; 2 Department of Human Studies, University of Alabama at Birmingham, Birmingham, AL 35294 USA

**Keywords:** Non-exercise activity thermogenesis (NEAT), Exercise training frequency, Older women

## Abstract

**Background:**

We have previously shown that 16 weeks of combined aerobic–resistance training in older women decreases non-exercise activity thermogenesis (NEAT) when performed 3 times/week, but not when performed once or twice weekly.

**Objectives:**

To examine changes from weeks 16 to 32 in anthropometrics, body composition, fitness, cytokines, mood, and energy expenditure (EE) components among training frequencies in older women.

**Methods:**

Sedentary women (60–74 y) previously randomized to 1 + 1, 2 + 2, or 3 + 3 sessions/wk of supervised combined aerobic–resistance training continued the program from weeks 16 to 32. Total EE (TEE) (doubly labeled water), resting EE (REE) (indirect calorimetry), training-related EE (TREE) (validated estimates), NEAT = activity EE (AEE) − TREE (AEE = 0.9×TEE − REE), fat mass (FM), and fat-free mass (FFM) (DXA), V̇O₂max (treadmill), mood/fatigue, and serum cytokines were measured at weeks 16 and 32. Two-way repeated-measures ANOVA was used with Tukey posthocs.

**Results:**

Fifty-seven women contributed anthropometry/fitness/cytokines; 44 contributed EE components. Weight decreased, whereas FM (kg and %), FFM, V̇O₂max, IL-6, CRP, TNF-α, fatigue, vigor and REE were unchanged. Bench- and leg-press strength increased across groups. Depression varied by group (lower in 3 + 3 vs. 2 + 2; *P =* 0.044). TEE differed between groups (*P =* 0.017; 2 + 2 > 1 + 1 and 3 + 3). TREE increased over time (*P =* 0.002) and differed by group (*P <* 0.001). NEAT showed a time-by-group interaction (*P =* 0.049), increasing only in 3 + 3 (*P =* 0.044).

**Conclusion:**

High-frequency (3 + 3) combined aerobic-resistance training was associated with increased NEAT between weeks 16–32, but not lower frequencies. However, the 2 + 2 group continued to have the highest NEAT overall.

**Supplementary Information:**

The online version contains supplementary material available at 10.1007/s00421-026-06161-5.

## Introduction

Aging is accompanied by changes in body composition and physical function, including decreases in fat-free mass (FFM) and strength, as well as increases in fat mass (FM), related in part to lower habitual physical activity (PA) and metabolic changes. These alterations matter clinically because diminished cardiorespiratory fitness and strength are associated with mobility limitation and mortality in older adults (Pahor et al. 2014). Combined aerobic and resistance training is recommended to counter these changes, but the time course and dose–response of adaptation past the first few months in older women has yet to be fully described (Westerterp 2017). Another characteristic of aging is low-grade systemic inflammation, also referred to as “inflammaging” and commonly indexed by circulating cytokines such as interleukin-6 (IL-6), C-reactive protein (CRP), and tumor necrosis factor-α (TNF-α) (Ferrucci and Fabbri 2018). Exercise has been demonstrated to attenuate these inflammatory markers; however, the effects of exercise vary by mode, frequency, intensity, and accompanying weight loss (Gleeson et al. 2011; Tayebi et al. 2025). Psychological well-being is also an important consideration in late life. Exercise training, especially with a resistance component, has been associated with reductions in depressive symptoms; however, the magnitude of change is not consistent across programs and populations (Schuch et al. 2016; Gordon et al. 2018).

Recent research suggests that total energy expenditure (EE) (TEE) may be constrained to the extent that increases in structured activity can be offset by reductions in EE elsewhere, implicating non-exercise activity thermogenesis (NEAT) as a potential site of compensation (Pontzer et al. 2016). The practical implications of this constrained model are that higher doses of exercise lead to energy deficits and magnitudes of weight loss that are smaller than predicted, due to compensation in other components of EE, as previously shown (Rosenkilde et al. 2012; Church et al. 2009).

Increasing training frequency might increase training-related EE (TREE) and accelerate fitness gains, but can also elicit behavioral or physiologic adaptations in the form of reduced NEAT or altered recovery that blunt changes in TEE and body composition. Additionally, the compensation might be time-dependent. Short-term studies in sedentary older women who undertook combined training in a controlled setting demonstrated significant strength and fitness improvements across all training frequencies, however, there were minimal additional benefits at the highest frequencies (Fisher et al. 2013). Longer-term training raises the question of whether activity-related compensation persists, decreases, or reverses over time. In mobility-limited older adults followed for 24 months in the LIFE program, the PA arm attenuated the reduction in overall activity, while shifting the individuals’ activity toward longer, higher-intensity bouts, suggesting that behavioral adaptation unfolds over extended periods (Wanigatunga et al. 2017). Other trials of months-long training have reported increased AEE without compensatory reductions in NEAT consistent with a late rebound after early reduction (Hollowell et al. 2009; Rangan et al. 2011; Willis et al. 2014).

Prior research by our group showed that over the initial 16 weeks, a training program two times/week (2 + 2) of combined aerobic-resistance training led to increases in NEAT and TEE, while a training program three times/week (3 + 3) led to a decrease in NEAT, even with comparable improvements in fitness, consistent with frequency-dependent behavioral compensation in older women (Hunter et al. 2013). However, we do not yet understand the longer-term response of NEAT to training beyond the initial 16 weeks of combined training. Similar studies in older adults indicated that resistance training can increase TEE and free-living activity depending upon the baseline level of PA and the type of program designed (Hunter et al. 2000; Wang et al. 2017).

To address this knowledge gap, we performed a secondary analysis on the long-term changes from weeks 16 to 32 in body weight (BW) and composition, cardiorespiratory fitness, strength, cytokines, mood, fatigue, and the components of EE, including TEE, REE, TREE, and NEAT. We were interested in understanding how training frequency may impact late-phase adaptations in the functional, inflammatory, psychological, and energetic domains. We hypothesized minimal or no changes in most variables (BW, FM and lean body mass (LBM), cytokine and mood responses, fitness and strength), but differences in EE patterns, with time- and group-specific effects such that, over time, TREE would continue to increase, whereas NEAT would diverge between groups.

## Methods

### Participants and study design

Older women (60–74 y), both European American (EA) and African American (AA), who were healthy, nonsmoking, sedentary (exercising < 1 time/week in the past year), free of metabolic diseases, and not taking medications with a known effect on EE were included in the study. The study was approved by the institutional review board and written informed consent was provided by all subjects. Measurements were obtained at baseline, week 16, and week 32, and this secondary analysis follows the same cohort through week 32. Seventy-four older women (60–74 year) were randomized at baseline to low-, moderate-, or high-frequency combined aerobic (A)-resistance (R) training performed on non-consecutive days: 1A + 1R (*n* = 26), 2A + 2R (*n* = 26), or 3A + 3R (*n* = 22). All completed a 16-week supervised combined aerobic and resistance program. The outcomes of these first 16 weeks have been previously published (Hunter et al. 2013). Fifty-seven women then continued their assigned regimens of 1A + 1R (*n* = 19), 2A + 2R (*n* = 20), or 3A + 3R (*n* = 18) through week 32.

### Exercise training

Fifty-minute training sessions were conducted in a laboratory environment and supervised by exercise physiologists. Every session began with 3–4 min on a cycle ergometer or treadmill, and ended with 3–4 min of stretching.

### Aerobic exercise

Participants exercised at ~ 80% HRmax for 40 min on each aerobic session. The exercise modes were cycle ergometer and treadmill, with ≥ 50% of the aerobic time using the treadmill.

### Resistance training

Exercises included leg press, squat, leg extension, leg curl, elbow flexion, lat pull-down, bench press, military press, lower-back extension, and bent-leg sit-ups. All exercises were performed for 2 sets × 10 repetitions at ~ 80% 1RM with resting between sets for 1.5–2 min. 1RM was measured every 5 weeks to track training loads and to verify the prescribed intensity. For more details about the training see Kalb and colleagues, 1991 (Kalb and Hunter 1991).

### Aerobic capacity

Maximal aerobic exercise testing was supervised by a physician using a modified Balke treadmill protocol (Beltz et al. 2016). Expired gases were measured using a calibrated metabolic cart (MAX-1; Physio-Dyne Instrument Corporation, Quogue, NY). Twelve-lead ECG was monitored continuously, and brachial blood pressure (BP) was measured every 2 min (Omron HEM-780; Omron Healthcare, Bannockburn, IL). The testing began with a 2-minute walk at 2 mph. Grade on the treadmill was then increased by 3.5% every 2 min until minute 12, after which grade was reduced to 12% and speed to 3 mph. Grade increased by 2.5% per minute until exhaustion. BP, heart rate, and oxygen uptake were taken during the last 20 s of each segment. Termination criteria met American Heart Association/American College of Cardiology guidelines (Gibbons et al. 1997, 2002). V̇O₂max (mL·kg⁻¹·min⁻¹), peak RER, and peak heart rate were set as the highest 20-s means. The maximum oxygen uptake was confirmed by ≥ 1 of: heart rate within 10 beats of age-predicted maximum, plateau in oxygen uptake, and/or RER ≥ 1.1.

### Resting energy expenditure (REE)

REE was taken between 06:00 and 08:00 AM after a 12-h fast. Subjects were awake, and measurements were taken in a quiet, dimly lit, and well-ventilated room at 22–24 °C. Subjects were recumbent on a bed with the head draped in a Plexiglas ventilated canopy. Post-training REE was taken ~ 41 h after the last resistance session. After a 15-min recovery period, REE was assessed for 30 min using open-circuit indirect calorimetry and a ventilated-canopy system (DeltaTrac II; SensorMedics, Yorba Linda, CA); the final 20 min were used for analysis. Continuously recorded oxygen consumption (V̇O₂) and carbon dioxide output (V̇CO₂) were averaged in 1-min increments. Energy expenditure and RER were calculated from V̇O₂ and V̇CO₂.

### Estimated energy cost of exercise training

Net oxygen consumption (exercise V̇O₂ - resting V̇O₂) was measured using a metabolic cart (MAX-1; Physio-Dyne, Quogue, NY) while walking on a grade that elicited a heart rate of ± 5 bpm above each subject’s training heart rate during the 2-wk post-training TEE (no training was performed during the pre-TEE test). V̇O₂ was sampled across minutes 0–5, 20–25, and 35–40, averaged, and expressed as kilocalories per 40-min aerobic session assuming 5 kcal·L⁻¹ O₂. For resistance exercise, we determined the cost of the workout + 15-min recovery in a subgroup of 25 subjects with a portable metabolic system (COSMED K4 b²; COSMED, Rome, Italy). From these measures, we derived a regression equation to estimate EE from training volume (amount of weight lifted per exercise), which we then cross-validated on an independent group of older women (*n* = 14) with R²=0.95 and SEE = 11 kcal using previously described techniques (Kalb and Hunter 1991). The measured values were used for the 25 instrumented subjects, the regression estimates to the remaining participants.

### Dual-energy X-ray absorptiometry (DXA)

Total FM and LBM were assessed by dual-energy X-ray absorptiometry (DXA) with a Lunar DPX-L densitometer (Lunar Radiation, Madison, WI) in the UAB Department of Nutrition Sciences. Scans were analyzed by adult software version 1.33. Arm FM was obtained by arm tissue mass (kg) multiplied by arm percent fat divided by 100. Leg FM was obtained in the same manner as above. Non-bone arm lean mass and leg lean mass were obtained by tissue mass (kg) multiplied by 1 minus percent fat divided by 100.

### Total energy expenditure (TEE)

TEE was assessed at baseline and at the end of the last 2 weeks of the intervention with doubly labeled water as previously described (Weinsier et al. 2002). The oral dose was 1 g·kg⁻¹ premix (10% H₂¹⁸O, 8% ²H₂O), and four urine samples were collected over a timed basis: two morning samples immediately after dosing and two samples 14 days later. Spaces for isotope dilution were derived by back-extrapolation of log enrichments to time zero; rCO₂ was derived from the Schoeller equation and TEE from rCO₂ using the Weir equation (assuming a food quotient), 1 mol CO₂ = 22.4 L (Schoeller et al. 1986; Weir 1949). Triplet samples for H₂¹⁸O and ²H₂O were measured by isotope-ratio mass spectrometry at the University of Alabama at Birmingham. In a reanalyzed subset (*n* = 7), daily values of TEE were highly reproducible (coefficient of variation 4.3%).

### Activity energy expenditure (AEE)

AEE was calculated as 0.9×TEE − REE. Reducing TEE by 10% was done to account for the thermic effect of food. NEAT was subsequently calculated as NEAT = AEE − energy cost of exercise training (Levine et al. 1999).

### Inflammatory markers

After an overnight fast and ≥ 40 h after the last exercise bout, venous blood was collected for analysis of cytokines. Inflammatory markers were measured by ELISA with high sensitivity in duplicate: TNF-α (Quantikine HSTA00C; R&D Systems, Minneapolis, MN), IL-6 (Quantikine HS600B; R&D Systems), and C-reactive protein (ALPCO 030–9710 s; Windham, NH).

### Perceptions of fatigue, vigor, and depression

Mood was assessed with the standard Profile of Mood States (POMS), a 65-item instrument with strong internal consistency and acceptable test–retest reliability (Leunes 2000). Participants rated each adjective on a 0–4 scale (“not at all” to “extremely”) based on how they felt during the past week. Although POMS provides a Total Mood Disturbance score based on six subscales (tension–anxiety, depression, anger–hostility, vigor–activity, fatigue, confusion–bewilderment), the analyses of this paper utilized the vigor, fatigue, and depression subscales only.

### Statistical analysis

The primary objective was to compare the impact of three training frequencies on TEE, AEE, and NEAT in older women. All results (except for age and height) were explored with two-way repeated-measures ANOVA with group factors (1A + 1R, 2A + 2R, 3A + 3R) and time (repeated). Age and height were compared by one-way (group) ANOVA. When there was a significant group or time-by-group effect, Tukey-adjusted post hoc analysis was applied to compare differences among groups and between-group contrasts at each time point. Statistical significance was set at α = 0.05. We also calculated Pearson correlation coefficients among change scores (week 32 − week 16) for pertinent variables to examine associations.

**Fig. 1:**
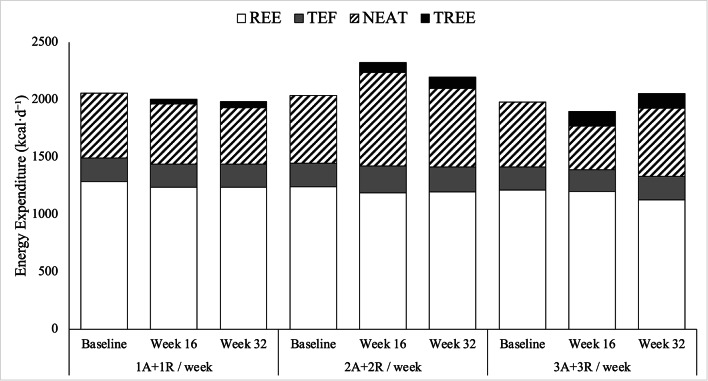
Energy-expenditure components at baseline, and at the end of 16 and 32 weeks of combined exercise training. TEE: Total energy expenditure; TREE: Training-related energy expenditure; NEAT: Non-exercise activity thermogenesis; REE: resting energy expenditure; TEF: thermic effect of feeding. TREE increased overall, while NEAT increased in the high-frequency group (3 + 3), declined in the moderate-frequency group (2 + 2), and changed little in the low-frequency group (1 + 1). TEE and REE showed no overall time effect

## Results

Fifty-seven women (10 AA) were included in this analysis, with an average age of 65.3 ± 3.4 years, and a BMI of 26.4 ± 4.4 kg·m⁻². Analytic sample sizes varied by outcome and time point. Anthropometric variables and fitness measures at weeks 16 and 32 across groups are shown in Table 1. There were no between-group differences in age or height. BW decreased modestly over time (*T =* 0.022), with no group or time-by-group effects. Total FM (kg and %), FFM, and arm and leg lean mass did not change significantly over time, and no time-by-group interactions were found (all *P* > 0.05). Maximal strength increased in the leg press and bench press (*T <* 0.001 and *T =* 0.003, respectively), with no time-by-group interactions. V̇O₂max did not exhibit significant time, group, or interaction effects (*T =* 0.452; *G =* 0.896; *T×G =* 0.259).

**Table 1:** Anthropometrics, body composition and fitness at baseline, weeks 16 and 32

	Low frequency (1A + 1R/week)			Moderate frequency (2A + 2R/week)			High frequency (3A + 3R/week)			*P*
	Baseline	Week 16	Week 32	Baseline	Week 16	Week 32	Baseline	Week 16	Week 32	
Age (yr)	65.6 ± 3.8 (*n* = 26)	65.8 ± 3.6 (*n* = 19)		64.2 ± 3.4 (*n* = 26)	64.9 ± 3.2 (*n* = 20)		64.6 ± 3.5 (*n* = 22)	65.1 ± 3.5 (*n* = 18)		*G = 0.709*
Height (cm)	167 ± 5.7 (*n* = 26)	167.1 ± 5.8 (*n* = 19)		165 ± 5.1 (*n* = 26)	165.8 ± 5.2 (*n* = 20)		164 ± 3.7 (*n* = 22)	164.1 ± 3.7 (*n* = 18)		*G = 0.241*
Weight (kg)	78.1 ± 14.3 (*n* = 26)	76.5 ± 13.7	75.2 ± 12.8 (*n* = 19)	74.2 ± 9.4 (*n* = 26)	73.9 ± 9.3	73.3 ± 9.1 (*n* = 20)	68.3 ± 9.6 (*n* = 22)	67.3 ± 10.3	67.2 ± 9.7 (*n* = 18)	T = 0.022* *G = 0.054* *T×G = 0.231*
% body fat	44.6 ± 6.4 (*n* = 26)	43.0 ± 8.0	42.0 ± 7.7 (*n* = 19)	43.3 ± 5.4 (*n* = 26)	41.5 ± 4.6	41.4 ± 5.2 (*n* = 20)	39.5 ± 6.3 (*n* = 22)	38.0 ± 7.0	38.1 ± 6.2 (*n* = 18)	*T = 0.144* *G = 0.102* *T×G = 0.104*
Fat mass (kg)	35.5 ± 11.4 (*n* = 26)	34.2 ± 12.0	33.0 ± 11.1 (*n* = 16)	32.4 ± 6.8 (*n* = 26)	31.2 ± 6.9	30.9 ± 7.6 (*n* = 14)	27.5 ± 7.4 (*n* = 22)	27.9 ± 7.7	28.0 ± 6.9 (*n* = 13)	*T = 0.076* *G = 0.257* *T×G = 0.161*
Fat-free mass (kg)	42.5 ± 4.2 (*n* = 26)	43.6 ± 4.6	43.7 ± 4.5 (*n* = 16)	41.8 ± 4.7 (*n* = 26)	43.5 ± 5.3	43.1 ± 4.4 (*n* = 14)	40.8 ± 3.4 (*n* = 22)	41.7 ± 4.1	41.9 ± 3.9 (*n* = 13)	*T = 0.821* *G = 0.524* *T×G = 0.282*
Leg press strength (kg)	90.9 ± 25.7 (*n* = 26)	110.6 ± 40.4	127.0 ± 49.1 (*n* = 13)	95.1 ± 20.6 (*n* = 26)	105.8 ± 23.4	115.9 ± 22.7 (*n* = 16)	93.5 ± 25.9 (*n* = 22)	118.9 ± 29.3	132.6 ± 42.1 (*n* = 10)	T < 0 0.001** *G = 0.549* *T×G = 0.601*
Leg lean mass (kg)	13.1 ± 1.6 (*n* = 21)	13.3 ± 2.0	13.4 ± 1.8 (*n* = 19)	13.1 ± 1.6 (*n* = 23)	13.3 ± 1.6	13.2 ± 1.4 (*n* = 20)	13 ± 1.3 (*n* = 20)	12.9 ± 1.5	12.9 ± 1.6 (*n* = 18)	*T = 0.939* *G = 0.682* *T×G = 0.261*
Bench press strength (kg)	22.2 ± 4.9 (*n* = 26)	25.4 ± 8.6	28.0 ± 7.2 (*n* = 14)	24.3 ± 6.3 (*n* = 26)	26.8 ± 6.9	30.1 ± 7.9 (*n* = 16)	24.2 ± 5.6 (*n* = 22)	28.5 ± 7.7	30.4 ± 6.2 (*n* = 11)	T = 0.003** *G = 0.606* *T×G = 0.794*
Arm lean mass (kg)	4.3 ± 0.7 (*n* = 21)	4.2 ± 0.7	4.2 ± 0.7 (*n* = 19)	4.3 ± 0.7 (*n* = 23)	4.3 ± 0.6	4.3 ± 0.6 (*n* = 20)	4.2 ± 0.5 (*n* = 20)	4.2 ± 0.7	4.2 ± 0.6 (*n* = 18)	*T = 0.062* *G = 0.918* *T×G = 0.912*
V̇O₂max (mL kg⁻¹ min⁻¹)	21.8 ± 4.5 (*n* = 24)	22.8 ± 5.2	23.9 ± 5.6 (*n* = 16)	22.6 ± 4.7 (*n* = 24)	24.3 ± 3.5	23.9 ± 4.0 (*n* = 17)	23.7 ± 4.3 (*n* = 21)	23.6 ± 6.0	23.8 ± 5.0 (*n* = 18)	*T = 0.452* *G = 0.896* *T×G = 0.259*

Values are presented as mean ± SD. *A :* aerobic, *R:* resistance. P values are based on repeated-measures ANOVA comparing outcomes at week 16 and week 32.

*p < 0.05, **p < 0.01

Cytokines and mood changes between weeks 16 and 32 are shown in Table 2. Cytokines (IL-6, CRP, TNF-α), as well as fatigue, and vigor did not change significantly over time, and no group or time-by-group interactions were seen (all *P* > 0.05). Depression differed by group (*G =* 0.041), without time or time-by-group effects. Post hoc analysis showed that the 3 + 3 group had lower depression than the 2 + 2 group (*P =* 0.044). These findings closely mirror the general fitness and body-composition pattern described in the first 16-week report, where strength improved while BW changed only modestly.

**Table 2:** Cytokines and mood (fatigue, depression, vigor) at baseline, weeks 16 and 32

	Low frequency (1A + 1R/week)			Moderate frequency (2A + 2R/week)			High frequency (3A + 3R/week)			*P*
	Baseline	Week 16	Week 32	Baseline	Week 16	Week 32	Baseline	Week 16	Week 32	
IL-6 (pg/mL)	2.0 ± 1.4 (*n* = 16)	1.7 ± 1.1	1.6 ± 0.8 (*n* = 17)	1.5 ± 0.6 (*n* = 18)	1.4 ± 0.6	1.4 ± 0.6 (*n* = 17)	2.4 ± 2.3 (*n* = 16)	2.3 ± 2.9	2.0 ± 2.1 (*n* = 16)	*T = 0.123* *G = 0.359* *T×G = 0.592*
C-reactive protein (mg/L)	2.5 ± 2.2 (*n* = 16)	2.6 ± 2.4	2.2 ± 2.5 (*n* = 15)	2.6 ± 3.0 (*n* = 18)	2.2 ± 2.3	2.4 ± 3.2 (*n* = 16)	3.7 ± 3.6 (*n* = 16)	3.9 ± 3.5	4.0 ± 4.2 (*n* = 16)	*T = 0.888* *G = 0.241* *T×G = 0.482*
TNF-α (pg/mL)	6.3 ± 2.1 (*n* = 16)	6.2 ± 2.2	6.6 ± 2.6 (*n* = 17)	6.9 ± 2.4 (*n* = 18)	6.8 ± 2.3	6.6 ± 2.0 (*n* = 17)	6.7 ± 2.3 (*n* = 16)	6.7 ± 2.4	6.3 ± 1.9 (*n* = 16)	*T = 0.858* *G = 0.937* *T×G = 0.087*
Fatigue	4.5 ± 6.0 (*n* = 20)	4.2 ± 3.8	4.8 ± 4.0 (*n* = 13)	4.7 ± 2.9 (*n* = 17)	4.9 ± 3.5	5.1 ± 2.1 (*n* = 13)	4.1 ± 3.8 (*n* = 18)	3.0 ± 3.2	4.1 ± 4.2 (*n* = 15)	*T = 0.171* *G = 0.479* *T×G = 0.693*
Depression	3.1 ± 4.4 (*n* = 20)	3.7 ± 4.3^**ab**^	1.8 ± 2.1^**ab**^ (*n* = 13)	2.9 ± 3.1 (*n* = 17)	5.1 ± 4.0**ᵃ**	4.5 ± 3.4**ᵃ** (*n* = 13)	2.9 ± 3.9 (*n* = 18)	2.1 ± 2.3^**b**^	2.6 ± 2.7^**b**^ (*n* = 15)	*T = 0.265* G = 0.041* *T×G = 0.242*
Vigor	19.4 ± 4.2 (*n* = 20)	20.2 ± 5.8	19.9 ± 5.7 (*n* = 13)	16.6 ± 4.9 (*n* = 17)	18.2 ± 5.0	17.8 ± 5.4 (*n* = 13)	19.4 ± 5.5 (*n* = 18)	20.4 ± 5.3	19.7 ± 5.6 (*n* = 15)	*T = 0.552* *G = 0.449* *T×G = 0.978*

Values are presented as mean ± SD. POMS subscales reported as raw scores. *A :* aerobic, *R:*= resistance.

P values are based on repeated-measures ANOVA with post hoc analyses comparing outcomes at week 16 and week 32.

*p < 0.05, **p < 0.01. For depression, values that do not share a superscript letter differ significantly between groups (Tukey post hoc, *P* < 0.05) (a > b)

EE results are summarized in Table 3 and illustrated in Fig. 1. Only forty-four women (8 AA) who had their NEAT measured at weeks 16 and 32 were included in the analysis of EE variables. No significant time, group, or time-by-group interaction was seen for REE (*T =* 0.222; *G =* 0.451; *T×G =* 0.149). No significant time effect (*T =* 0.991) or time-by-group interaction (*T×G =* 0.141) was seen for TEE, but TEE differed between groups (*G =* 0.017; post hoc: 2 + 2 > 1 + 1, *P =* 0.040; 2 + 2 > 3 + 3, *P =* 0.033). TREE increased over time (*T =* 0.002) and differed by group (*G <* 0.001), with no time-by-group interaction (*T×G =* 0.124). Post hoc analysis revealed that the 2 + 2 group had a higher TREE than 1 + 1, and 3 + 3 had a higher TREE than 2 + 2 (both *P <* 0.001).

**Table 3:** TEE, REE, TREE, and NEAT at baseline, weeks 16 and 32

	Low frequency (1A + 1R/week)			Moderate frequency (2A + 2R/week)			High frequency (3A + 3R/week)			*P*
	Baseline (*n* = 26)	Week 16	Week 32 (*n* = 15)	Baseline (*n* = 26)	Week 16	Week 32 (*n* = 16)	Baseline (*n* = 22)	Week 16	Week 32 (*n* = 13)	
REE (kcal·d⁻¹)	1286 ± 212	1236 ± 132	1239 ± 155	1241 ± 167	1189 ± 152	1195 ± 188	1214 ± 144	1201 ± 196	1128 ± 160	*T = 0.222* *G = 0.451* *T×G = 0.149*
TEE (kcal·d⁻¹)	2057 ± 421	2006 ± 365^**b**^	1982 ± 258^**b**^	2038 ± 404	2325 ± 411**ᵃ**	2195 ± 391**ᵃ**	1978 ± 361	1897 ± 285^**b**^	2053 ± 339^**b**^	*T = 0.991* G = 0.017* *T×G = 0.141*
TREE (kcal·d⁻¹)	-	42 ± 8**ᶜ**	53 ± 9**ᶜ**	-	86 ± 10^**b**^	97 ± 18^**b**^	-	125 ± 9**ᵃ**	125 ± 15**ᵃ**	T = 0.002** G < 0.001** *T×G = 0.124*
NEAT (kcal·d⁻¹)	565 ± 270	527 ± 315^**b**^	492 ± 236^**b**^	593 ± 345	817 ± 373**ᵃ**	683 ± 337**ᵃ**	567 ± 314	381 ± 223^**b**^	594 ± 202^**b†**^	*T = 0.791* G = 0.004** T×G = 0.049*

Values are presented as mean ± SD. *A :* aerobic, *R :* resistance. *REE*: Resting Energy Expenditure, *TEE*: Total Energy Expenditure, *TREE*: Training-Related Energy Expenditure. *NEAT*: Non-Exercise Activity Thermogenesis.

P values are based on repeated-measures ANOVA with post hoc analyses comparing outcomes at week 16 and week 32.

*p < 0.05, **p < 0.01. Within each EE variable, values that do not share a superscript letter differ significantly (a > b > c) (Tukey post hoc, *P <* 0.05). † Significant change from week 16 within group (Tukey post hoc, *P =* 0.044)

No time effect was observed for NEAT, but there was a significant group effect (*G =* 0.004), with post hoc analysis showing that the 2 + 2 group had higher NEAT than groups 1 + 1 and 3 + 3 (*P =* 0.014 and *P =* 0.010). There was a significant time-by-group interaction (*P =* 0.049), with post hoc analysis showing that the group 3 + 3 increased NEAT (*P =* 0.044), whereas neither 1 + 1 nor 2 + 2 changed significantly. Directionally, NEAT increased in the high-frequency group (~ + 213 kcal·d⁻¹), decreased in the moderate-frequency group (~ − 134 kcal·d⁻¹), and changed little in the low-frequency group (~ − 35 kcal·d⁻¹).

Correlation analyses among change scores (Table 4) showed that NEAT strongly tracked with TREE (*r =* 0.943, *P <* 0.01), suggesting that higher training-related EE was associated with higher free-living activity between weeks 16 and 32. Changes in NEAT were not significantly associated with changes in strength, cytokines or body composition variables. V̇O₂max changes were inversely correlated with IL-6 (*r = −* 0.406, *P <* 0.01) and were positively correlated with depression (*r =* 0.400, *P <* 0.05). Depression also correlated inversely with leg-press strength (*r = −* 0.451, *P <* 0.05).

**Table 4:** Pearson correlation coefficients across variables of interest

All variables denote change scores (Δ = week 32 − week 16)	V̇O₂max	TREE	Bench-press strength	Leg-press strength	Leg lean mass	Depression score	Vigor score	IL-6	CRP	TNF-α
TREE	0.329* (39)									
Bench-press strength	−0.101 (36)	0.238 (32)								
Leg-press strength	−0.012 (34)	−0.037 (31)	0.126 (40)							
Leg lean mass	0.039 (51)	−0.145 (43)	0.112 (41)	0.056 (39)						
Depression score	0.400* (38)	0.134 (33)	−0.219 (28)	−0.451* (28)	−0.132 (41)					
Vigor score	0.055 (38)	0.006 (33)	−0.077 (28)	−0.039 (28)	0.081 (41)	−0.073 (41)				
IL-6	−0.406** (44)	−0.332* (37)	−0.008 (35)	0.047 (33)	0.154 (50)	−0.150 (36)	−0.063 (36)			
CRP	−0.080 (43)	0.089 (37)	−0.103 (35)	−0.270 (33)	−0.114 (49)	0.014 (35)	0.112 (35)	−0.044 (50)		
TNF-α	−0.193 (43)	−0.282 (36)	0.035 (35)	−0.339 (33)	0.076 (49)	−0.087 (35)	0.039 (35)	0.095 (50)	0.039 (49)	
NEAT	0.300 (39)	0.943** (38)	0.102 (34)	−0.091 (32)	−0.120 (44)	0.101 (31)	0.083 (31)	−0.234 (39)	0.032 (39)	−0.179 (38)

Values are presented as: r (N), *p < 0.05, **p < 0.01. *TREE*: Training-related energy expenditure, *NEAT*: Non-exercise activity thermogenesis, *IL-6*: Interleukin-6, *CRP*: C-reactive protein, *TNF-α*: Tumor necrosis factor-α

## Discussion

In this extended phase of combined aerobic and resistance training from weeks 16 to 32, older women in our sample continued to adapt in physiological and behavioral ways. BW decreased significantly, even if mildly, even though participants were not asked to lose weight. At the same time, overall body composition remained relatively stable. FM (kg and %) and FFM did not change over time in any of the training frequency groups, and FFM was generally maintained rather than being lost. This is particularly important for older women who are susceptible to lean tissue loss as they age (Bamman et al. 2003). Thus, the fact that weight decreased while FFM remained constant indicates that strength-related tissue was maintained by the training regimen, even in the absence of significant muscle growth. Furthermore, strength metrics of leg press and bench press both increased from week 16 to 32 in all three frequency groups, supporting the hypothesis that continued exposure to progressive resistance continued to yield functional benefits well past the initial 16-week period. However, arm and leg lean mass did not change significantly during this time period. This suggests that late-phase gains in strength are likely attributed primarily to neural and skill adaptations such as motor unit recruitment, coordination, and exercise economy rather than hypertrophy (Sale 1988; Folland and Williams 2007). Aerobic capacity did not change significantly over this same period, suggesting that most of the cardiorespiratory gains probably occurred earlier in the training period (first 16 weeks) and then plateaued.

Stress and recovery markers were generally stable. Inflammatory cytokines such as IL-6, CRP and TNF-α did not change significantly, and feelings of fatigue and vigor did not worsen with ongoing training. Mood also seemed to change in a potentially significant manner. Although overall changes with time were not significant, depression scores were lower in the high-frequency group compared to the moderate-frequency group. This is opposite to the conventional overtraining model in athletes, where high exercise volume is usually linked with more depressive symptoms, mediated at least partly by inflammatory signaling. While earlier studies showed that blocks of high-intensity training can worsen mood symptoms in competitive swimmers (Morgan et al. 1988; Raglin et al. 1991), in older adults, the right dose of resistance training has been associated with improved mood and no chronic inflammatory elevation (Gordon et al. 2018; Schuch et al. 2016). In a 24-week trial of sedentary older adults exercising three times per week, McLafferty and colleagues reported that resistance training improved several Profile of Mood States subscales, including Confusion, Tension, and Anger and Total Mood Scores (McLafferty et al. 2004). Our results are more consistent with the latter pattern, suggesting that, at least for some women, maintaining a high training frequency was not associated with psychological stress but with a more favorable affective profile. Taken together, all these results are indicative of a generally acceptable and favorable adaptation to extended combined training.

Regarding EE, our findings indicate that, during the weeks 16–32 phase of combined aerobic-and-resistance training in older women, TREE increased, while NEAT trajectories diverged by training frequency. NEAT increased in the high-frequency group (3 + 3), decreased in the moderate-frequency group (2 + 2), and remained fairly stable in the low-frequency group (1 + 1). TEE and REE showed no overall time effects. Although the exercise prescription and frequency allocation were designed to differ among groups (1 + 1, 2 + 2, 3 + 3), the distribution of aerobic and resistance training was held constant within each group for the entire duration of the intervention, as established through randomization. All training sessions were closely supervised to promote adherence to the aerobic and resistance exercise protocols. Thus, the between-group differences in TREE largely reflect the assigned weekly training dose rather than changes in modality distribution over time. Additionally, body composition was relatively stable between weeks 16 and 32, with no significant time, group, or time by group effects for FM or FFM, indicating that the changes in EE components were not driven by concurrent changes in body size or composition. These results extend our original 16-week findings which showed increased TEE, AEE and NEAT in the 2 + 2 group, positive trend for NEAT in 1 + 1 group, and a substantial decrease in NEAT in the 3 + 3 group (Hunter et al. 2013), by demonstrating a late-phase rebound of NEAT in the high-frequency group, suggesting a physiological and/or behavioral adaptation to the high-training frequency.

One reason for our findings might be time-dependent behavioral adaptations. During initial training, high frequency may displace free-living activity due to fatigue, soreness, or time constraints, which we actually speculated in our original 16-week report (Hunter et al. 2013). However, with continued exposure, participants restructure their daily routines and restore spontaneous activity. Longitudinal accelerometry in mobility-limited older adults in the LIFE study supports gradual change in activity patterns towards greater length and more intense bouts (Wanigatunga et al. 2017). The attenuation of total-activity decline over 24 months is consistent with gradual behavioral adaptation rather than immediate compensation set points. The results from this cohort align with our late rebound between weeks 16 and 32, though on a shorter timescale.

In terms of TEE, our initial 16-week findings align with the constrained model. Pontzer and colleagues suggested that as structured exercise increases, the body conserves energy elsewhere, so TEE does not rise linearly with dose (Pontzer et al. 2016). Prior studies show that compensation often occurs via reductions in non-exercise activity or other physiological costs. In a 13-week trial, Rosenkilde and colleagues likewise found that moderate and high aerobic doses produced similar FM loss, indicating offsets outside the workout window (Rosenkilde et al. 2012). Consistent with this, Church and colleagues reported in a six-month dose–response trial that higher exercise volumes led to less weight loss than predicted (Church et al. 2009). Our data suggests that this reduction may be transient in older women exposed to high frequency of combined training, with NEAT recovering by week 32 as training became part of their daily life.

Our results are in line with longer-term studies suggesting that activity outside training can be maintained or even increase over time. Hollowell and colleagues reported increased total AEE with no reduction in non-exercise AEE over an eight-month period (Hollowell et al. 2009). Rangan and colleagues also noted that eight months of combined aerobic and resistance training increased AEE without compensatory reductions in NEAT (Rangan et al. 2011). Additionally, Willis and colleagues reported over a period of ten months that NEAT and non-exercise PA did not decline on average despite ongoing aerobic sessions (Willis et al. 2014). With respect to resting metabolism, most exercise trials show little fluctuation in REE, suggesting that compensation is concentrated in activity or other costs rather than at rest (Fernández-Verdejo et al. 2021). In our study, REE did not differ overall while free-living activity varied by group. This pattern of a stable REE with frequency-dependent shifts in NEAT fits the constrained-TEE model, in which energy partitioning rather than TEE changes during training. Together, these findings suggest that the location and duration of energy conservation in the body are dynamic and may shift over periods of weeks to months. Early reductions in free-living PA, particularly in response to higher-frequency training, may represent a transient adaptation. Over time, as individuals adjust, and reestablish daily routines, spontaneous PA may recover.

The correlation analysis revealed a strong positive association between NEAT and TREE, implying increased TREE was associated with increased free-living activity between weeks 16 and 32. This is in agreement with the previously mentioned studies reporting that longer-duration programs can increase total activity, without reducing non-exercise activity, and further supports the late-phase NEAT rebound we identified (Hollowell et al. 2009; Rangan et al. 2011; Willis et al. 2014). The absence of any other significant correlation between NEAT and changes in strength, LBM, or cytokines supports that the adaptation in free-living activity during the late phase was primarily through behavioral mechanisms rather than muscle or inflammatory adaptations. The negative correlation between V̇O₂max and IL-6 is consistent with previous work suggesting that higher cardiorespiratory fitness is associated with lower systemic inflammation (Beavers et al. 2010; Petersen and Pedersen 2005). Additionally, the negative association between depression and leg-press strength aligns with previous research suggesting that greater muscular fitness is positively tied with better mood in older populations (Singh et al. 2005; Netz et al. 2005). Finally, the positive correlation between V̇O₂max and depression should be interpreted cautiously due to small sample size and multiple testing. Taken together, these results suggest a complex interaction between PA, inflammation, and mood, with both behavior and physiology influencing adaptations to prolonged training.

Strengths of this study include its longitudinal design that follows the same women from weeks 16 to 32. This extended time window enabled us to observe later-stage adaptations in free-living behavior, such as the rebound of NEAT in the highest-frequency group. Another strength is the randomized allocation to three different training frequencies, which allowed us to attribute differences in NEAT and mood to frequency per se and not to self-selected behavior. The study also combines tightly supervised exercise training with gold-standard physiological measurements. TEE was measured with doubly labeled water, REE with indirect calorimetry, TREE with directly measured and validated cost estimates, and body composition with DXA. In addition, we evaluated a number of pertinent domains such as strength, aerobic capacity, inflammatory cytokines, and mood rather than a single outcome, hence providing a more complete picture of older women’s tolerance to prolonged combined training with different frequencies. Finally, by partitioning TEE into resting, training-related, and NEAT, and tracking how those components shifted with time and frequency, the current study provides a direct insight into how physiological compensation unfolds in older women assigned to different training frequencies.

However, our study also has some limitations. First, the results apply specifically to relatively healthy, sedentary, 60–74-year-old women who were able to complete 16 weeks of combined aerobic and resistance training and maintained the same frequency allocated through week 32. Second, every exercise session was supervised and performed in a controlled laboratory setting, which required subjects to come to the facility multiple times per week. The structure, accountability, and social interaction provided by that setting potentially maintained adherence, recovery routines, and mood in a way that may not be universally accomplished in a home-based or unsupervised program. Third, not every outcome was available for every participant at weeks 16 and 32, particularly for EE variables. Although mixed-model analysis can accommodate unbalanced data, this still introduces the possibility that those who provided complete EE data may be systematically different in their behavior or physiology from others who did not. Importantly, the NEAT was calculated based on the standard partitioning formula (NEAT = AEE – TREE; AEE = 0.9× TEE – REE). Although this is a widely accepted formula, the NEAT calculated via this methodology represents the residual component of non-training energy expenditure and could indicate not just movement-based non-exercise activity, but also any potential alterations within the remaining parts of unmeasured energy expenditure such as sleeping metabolic rate, thermoregulatory expenditure, circadian variation in resting metabolism, or stress-induced alterations in metabolic rate. Thus, changes in the derived NEAT should indicate alterations in non-training energy activity rather than as an objective measurement of the relative amount of physical activity being performed. We were unable to compare movement-related activity patterns within this cohort to the calculated NEAT responses due to the absence of objective measures of activity such as accelerometry or activity logs. Finally, our follow-up did not extend beyond 32 weeks, so whether the rebound in NEAT we saw in the highest-frequency group would persist, amplify, or eventually regress toward baseline activity with increased duration remains unclear.

Collectively, our results suggest that compensation is dynamic. In older women, high-frequency exercise can replace free-living PA at first but, with continued exposure, can eventually improve with return of spontaneous daily activity. This suggests that prescribing combined aerobic and resistance training at a more frequent dosage is well tolerated in the long term. By 32 weeks, older women exercising three times per week in each modality showed preserved resting metabolism, increased strength, lower depression scores, and a rebound in NEAT. These results suggest that high-frequency combined training is feasible and behaviorally appropriate in the long term in older women.

## Supplementary Information

Below is the link to the electronic supplementary material.

Supplementary Material 1

## Data Availability

Available from the corresponding author upon reasonable request.
