# Supplementary material for: Frequency-dependent adaptations in energy expenditure, strength, mood, and inflammation following 16 to 32 weeks of combined aerobic and resistance training in older women
Source: Eur J Appl Physiol. 2026 Mar 24;126(7):3763–74. doi: 10.1007/s00421-026-06161-5 (PMC13380546; doi:10.1007/s00421-026-06161-5)
Supplement: Supplementary file 1 — Supplementary Material 1 [file 421_2026_6161_MOESM1_ESM.docx]

**Dear Editor,**

We are pleased to submit the revised version of our manuscript entitled **“Frequency-Dependent Adaptations in Energy Expenditure, Strength, Mood, and Inflammation following 16 to 32 Weeks of Combined Aerobic and Resistance Training in Older Women”** for consideration in the European Journal of Applied Physiology. We would like to take this opportunity to express our appreciation to the reviewers for the excellent suggestions, constructive comments and careful evaluation of our work, which have helped us to make significant improvements to the manuscript.

In response to the reviewers’ feedback, we have **(1)** added baseline values to the main tables and Figure 1 to provide fuller context across the entire intervention period, **(2)** added a statement discussing the limitations of the NEAT variable, stating that changes in NEAT could also be attributed to changes in other components of the energy expenditure measurement that are not measured directly, **(3)** added clarification regarding the stability of body size and composition during weeks 16–32, and **(4)** clarified that the aerobic-to-resistance training distribution was fixed within each randomized group across the intervention, such that differences in TREE reflect the assigned training dose.

Please find attached the detailed point-by-point responses to reviewer comments and a modified copy of the manuscript showing changes for the reviewers’ convenience. Thank you for your time and consideration. We look forward to hearing from you.

**Best regards,**
Abdelrahman M Elshakankiry, Department of Nutrition Sciences, University of Alabama at Birmingham (UAB), Birmingham, AL 35294-3360, USA. (Email: [amelshak@uab.edu](mailto:amelshak@uab.edu)) (On behalf of all authors).
